# The m^6^A Reader YTHDC2: Molecular Mechanisms and Regulatory Networks in Disease Pathogenesis

**DOI:** 10.3390/cells15161467

**Published:** 2026-08-16

**Authors:** Yanying Hu, Qi Zhou, Ning Xu, Ning Du, Shuangping Yang, Shiyan Gu

**Affiliations:** Institute of Preventive Medicine, School of Public Health, Dali University, No. 22, Wanhua Road, Dali 671000, China; hyy0881@163.com (Y.H.);

**Keywords:** YT521-B Homology (YTH) Domain Containing 2, N^6^-methyladenosine (m^6^A) modification, nervous system, respiratory system diseases, digestive system diseases, reproductive system, immune system

## Abstract

As a key N^6^-methyladenosine (m^6^A)-binding protein, YT521-B Homology (YTH) Domain-Containing Protein 2 (YTHDC2) plays a central role in the epitranscriptomic regulatory network. This protein specifically recognizes and binds to m^6^A modification sites on RNA molecules through its highly conserved YTH domain. This recognition exhibits high selectivity and affinity, thereby enabling precise control over the fate of target RNAs. At the molecular level, YTHDC2 is widely involved in various stages of the RNA life cycle, including core biological processes such as RNA splicing and processing, nuclear–cytoplasmic transport, translational efficiency regulation, and RNA decay. In recent years, accumulating evidence indicates that YTHDC2 participates in a variety of pathophysiological processes in an m^6^A-dependent manner. However, the robustness of evidence regarding YTHDC2 is heterogeneous across disease contexts. While certain pathologies are supported by rigorous mechanistic validation, others rely primarily on expression correlations or bioinformatic analyses. This review systematically synthesizes current knowledge regarding the multifaceted roles of YTHDC2 in disease progression, prognosis, and therapy, offering a comprehensive framework to guide future investigations.

## 1. Background

N^6^-methyladenosine (m^6^A) is an RNA methylation modification that occurs at the N^6^ position of adenine and plays critical roles in various pathophysiological processes. YTH domain-containing protein 2 (YTHDC2), also known as ATP-dependent RNA helicase YTHDC2, is a member of the YTH protein family and contains both a conserved YTH domain and translation-associated RNA helicases [1]. A recent review has demonstrated that YTHDC2 modulates various stages of mRNA metabolism, such as splicing, nuclear export, decay, and translation [2]. Through m^6^A recognition, YTHDC2 binds specific transcripts to either boost translation or facilitate degradation [3]. Mechanistically, YTHDC2 promotes translation by activating its RNA helicase activity to resolve secondary structures impeding elongation, and by binding 18S rRNA to anchor target mRNAs to the ribosome. Conversely, YTHDC2 interacts with 5′-3′ exoribonuclease 1 (XRN1) to form a complex that mediates target mRNA degradation, reduces stability, and suppresses gene expression [4,5].

Importantly, whether YTHDC2 promotes translation or accelerates decay is not predetermined, but rather dictated by the specific target mRNA and its cellular context [4,5]. The position of m^6^A modifications critically dictates YTHDC2 function. Specifically, m^6^A sites within the coding sequence (CDS) preferentially drive YTHDC2-mediated translational enhancement via helicase activity, whereas 3′ untranslated region (3′ UTR) sites are typically linked to mRNA stability regulation via XRN1 recruitment [6]. Furthermore, the downstream consequences of YTHDC2 depend on its interacting partners: associations with XRN1, UPF1 RNA helicase and ATPase (UPF1), or Moloney leukemia virus 10 protein (MOV10) promote degradation, while engagement with 18S rRNA facilitates translation [6]. Subcellular localization also contributes to functional specificity, as nuclear YTHDC2 primarily mediates RNA processing and splicing, whereas cytoplasmic YTHDC2 participates in translation and decay [4,6]. Beyond classical mRNA regulation, YTHDC2 modulates long non-coding RNA networks and broadly orchestrates diverse cellular processes, including ferroptosis, autophagy, immune signaling, and metabolic reprogramming [7,8,9,10,11]. Importantly, these regulatory effects exhibit striking context-dependent duality across different pathophysiological settings, allowing YTHDC2 to fine-tune the balance between transcript stability and translation to either promote or restrict disease progression [12,13]. Furthermore, cell type and microenvironment dictate the repertoire of target transcripts and interacting partners, thereby driving the context-dependent outcomes observed across tissues [14]. Collectively, m^6^A location, protein interactions, subcellular localization, and cellular context coordinately govern whether YTHDC2 drives the translation or degradation of target mRNAs. Such functional versatility is particularly evident when examining the roles of YTHDC2 in both physiological and pathological states [7]. In normal physiological settings, YTHDC2 participates in fundamental processes such as neurogenesis, meiotic progression, and metabolic homeostasis by stabilizing or destabilizing distinct sets of tissue-specific transcripts [5,15,16]. However, this same molecular machinery can be co-opted in disease states, where YTHDC2 exhibits striking context-dependent and tissue-specific roles. For example, YTHDC2 promotes the malignant progression of breast cancer by enhancing tumorigenesis and metastasis [17], yet its high expression in tumor-associated macrophages predicts favorable outcomes in triple-negative breast cancer by indicating anti-tumoral macrophage polarization [18]. Such functional duality underscores that YTHDC2 does not exert a uniform effect across different tissues. Rather, its role is dictated by the repertoire of m^6^A-modified target genes expressed in each cell type and the local molecular microenvironment. Thus, understanding both the core machinery of YTHDC2-mediated RNA regulation and its tissue-specific target networks is essential for interpreting its diverse contributions to disease pathogenesis.

This review provides the first systematic and comparative synthesis of YTHDC2 functions across human diseases. Specifically, we examine tumorigenic and non-tumorigenic conditions affecting multiple organ systems. By integrating mechanistic evidence with correlative findings within a broad comparative framework, this review aims to offer novel perspectives on YTHDC2 biology and its diagnostic and therapeutic potential.

## 2. Multifaceted Roles of YTHDC2 in Nervous System Diseases

Emerging evidence highlights YTHDC2 as a crucial regulatory factor mediating the onset and progression of neurological disorders. In the context of acute brain injury, YTHDC2 mitigates secondary damage following intracerebral hemorrhage (ICH) by reducing nuclear receptor coactivator 4 (NCOA4) mRNA stability in an m^6^A-dependent manner, thereby decreasing NCOA4-mediated ferritinophagy [19]. Similarly, during cerebral ischemia/reperfusion (I/R) injury, YTHDC2 modulates ferroptosis through its interaction with Methyltransferase-like protein 3 (METTL3)-modified lncRNA AU020206, which subsequently regulates Solute Carrier Family 7 Member 11 (SLC7A11) mRNA stability and glutathione peroxidase 4 (GPX4) expression. Disrupting this axis exacerbates neuronal injury and iron overload, whereas restoring SLC7A11/GPX4 confers neuroprotection [20]. Beyond ferroptosis, YTHDC2 contributes to temporal lobe epilepsy by mediating SLC7A11-dependent glutamate dysregulation in astrocytes. Mechanistically, it promotes the m^6^A-dependent translation of SLC7A11 mRNA, leading to cystine/glutamate antiporter overexpression and elevated extracellular glutamate, which exacerbates seizure activity [21]. Furthermore, YTHDC2 orchestrates neuroinflammatory responses following spinal cord injury (SCI). It upregulates lnc-Gstm5 via m^6^A modification, which recruits suppressor of variegation 3-9 homolog 1 (SUV39H1) to increase histone H3 lysine 9 trimethylation (H3K9me3) levels and inhibit nuclear factor-κB (NF-κB) signaling, ultimately mitigating inflammation [22]. Notably, YTHDC2 also exerts critical functions in neurodevelopment and repair. It enhances the stability and translation of low-density lipoprotein receptor-related protein 2 (Lrp2) mRNA to promote hippocampal neurogenesis, potentially alleviating memory impairment and depressive behaviors [15]. Conversely, YTHDC2 suppresses the neural differentiation of human embryonic stem cells by recruiting ten-eleven translocation 1 (TET1) to long terminal repeat 7/human endogenous retrovirus H (LTR7/HERV-H) genomic loci via m^6^A-modified HERV-H RNAs, thereby preventing epigenetic silencing of this transposable element [23].

In summary, YTHDC2 acts as a versatile therapeutic target across diverse neurological disorders by orchestrating m^6^A-dependent networks governing neurodevelopment, ferroptosis, excitotoxicity, and neuroinflammation. However, its role in nervous system tumors remains unexplored. Elucidating this gap in future studies will offer novel perspectives for managing these malignancies.

## 3. Multifaceted Roles of YTHDC2 in Respiratory Diseases

Respiratory diseases, including nasopharyngeal carcinoma, lung cancer, and pulmonary tuberculosis, remain a significant global health burden due to their persistently high incidence and mortality rates.

### 3.1. Mechanisms of YTHDC2 in Respiratory Tumors

Current evidence indicates that YTHDC2 exerts context-dependent, predominantly oncogenic roles in respiratory malignancies. In nasopharyngeal carcinoma (NPC), YTHDC2 is significantly upregulated and drives radioresistance by enhancing m^6^A-dependent insulin-like growth factor 1 receptor (IGF-1R) mRNA translation. This activates the IGF-1R/AKT/S6 axis, conferring a survival advantage under radiation stress [24]. In non-small cell lung cancer (NSCLC), YTHDC2 modulates the oncogenic lncRNA ZNRD1-AS1 via m^6^A modification, which subsequently promotes malignant proliferation, migration, and angiogenesis through the miR-942/Tensin 1 (TNS1) axis [25]. Moreover, YTHDC2 cooperates with alkB homolog 5, RNA demethylase (ALKBH5) to regulate the m^6^A-modified lncRNA CALML3-AS1, which recruits Enhancer of Zeste 2 Polycomb Repressive Complex 2 Subunit (EZH2) to methylate and epigenetically silence BTNL9, further driving NSCLC progression [26]. Notably, m^6^A modification at the A856 site within the vascular endothelial growth factor A (VEGFA) 5′UTR Internal Ribosome Entry Site-A (IRES-A) recruits the YTHDC2/Eukaryotic Translation Initiation Factor 4 Gamma 1 (eIF4GI) complex, enhancing cap-independent VEGFA translation to stimulate angiogenesis and lung tumor growth [27]. Collectively, these findings underscore the oncogenic function of YTHDC2 in NPC and specific NSCLC contexts. In contrast, YTHDC2 exhibits tumor-suppressive activities in lung adenocarcinoma (LUAD). Mechanistically, YTHDC2 destabilizes Mitochondrial Ribosomal Protein L12 (MRPL12) mRNA in an m^6^A-dependent manner, thereby repressing LUAD cell growth and mobility in vitro and tumor growth in vivo [28]. Furthermore, YTHDC2 downregulation decreases the expression of 2′,5′-oligoadenylate synthetase (OAS) and interferon-induced protein with tetratricopeptide repeats (IFIT) family members. The inhibition of these factors enhances exosome content, indicating that YTHDC2 normally restricts oncogenic exosome-mediated intercellular communication [29]. Additionally, YTHDC2 modulates macrophage polarization within the tumor microenvironment. By inhibiting cell division cycle-associated protein 4 (CDCA4), YTHDC2 overexpression increases the M1/M2 ratio and suppresses tumor growth, thereby fostering an anti-tumor immune microenvironment in LUAD [30].

**Figure 1:**
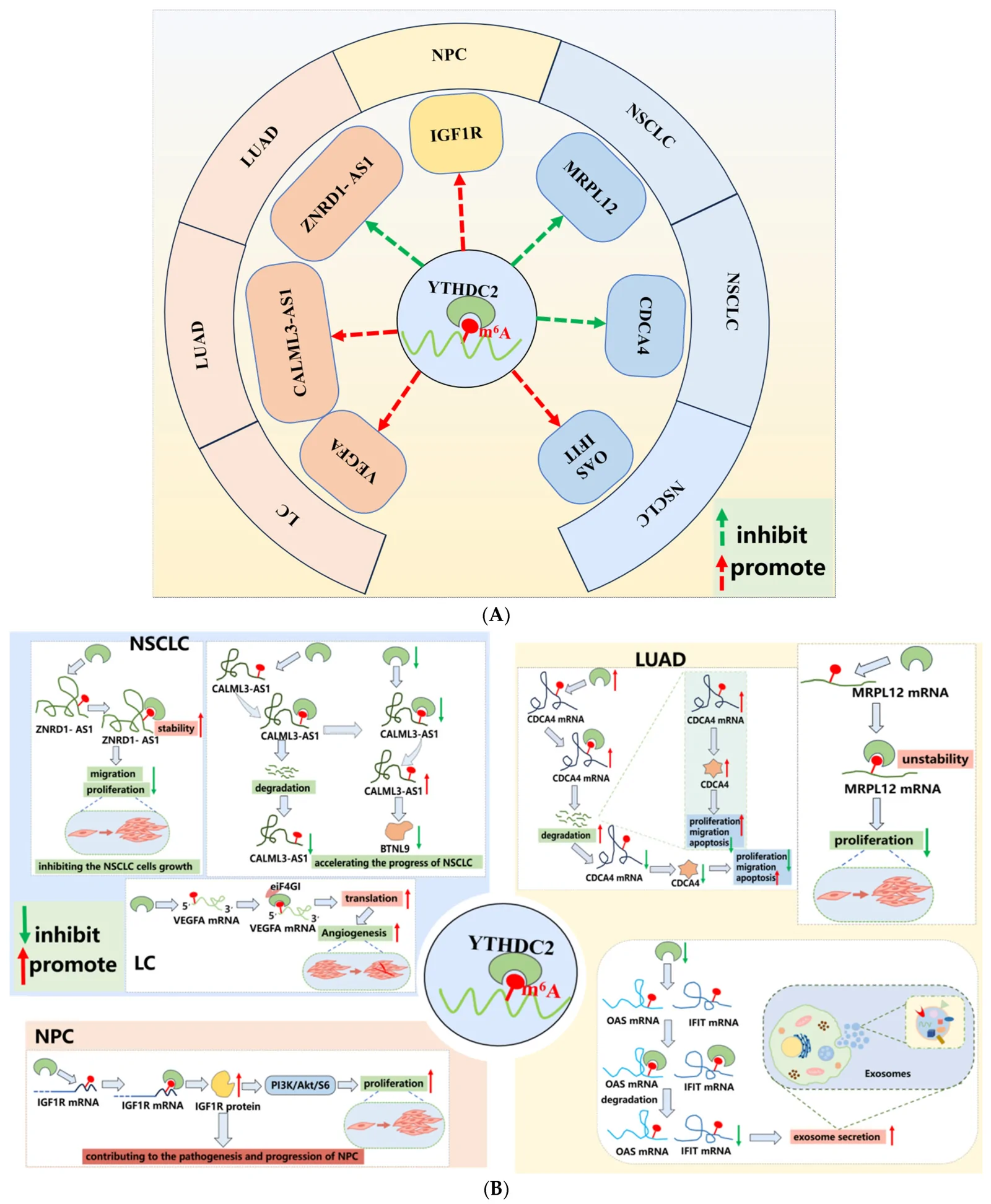
m^6^A-dependent mechanisms of the reader YTHDC2 in three respiratory system tumors (**A**) Schematic diagram of the functional modes: YTHDC2 primarily exerts a tumor-promoting role in nasopharyngeal carcinoma (NPC), whereas in non-small cell lung cancer (NSCLC) and lung adenocarcinoma (LUAD), its effect depends on the specific target genes it regulates and may exhibit either tumor-promoting or tumor-suppressing functions. (**B**) Distinct dual mechanisms of YTHDC2 in three respiratory tumors. YTHDC2 drives NPC progression via the IGF1R/PI3K/AKT cascade. It exerts bidirectional effects in NSCLC through ZNRD1-AS1, CALML3-AS1 and VEGFA. In LUAD, YTHDC2 oppositely modulates CDCA4 and MRPL12, and accelerates OAS/IFIT mRNA degradation to boost exosome secretion.

Altogether, YTHDC2 functions are highly subtype-dependent in respiratory tumors. It acts as an oncogene in NPC and exhibits dual effects in NSCLC, with pro-tumorigenic pathways such as VEGFA and ZNRD1-AS1 being well characterized. In contrast, it primarily serves as a tumor suppressor in LUAD. Given current study heterogeneity, large-scale cohorts are warranted to elucidate the dominant functional axes and draw definitive conclusions for each subtype. The multifaceted roles of YTHDC2 in respiratory tumors are summarized in Table 1. And m^6^A-dependent mechanisms of the reader YTHDC2 in three respiratory system tumors were represented in Figure 1.

### 3.2. Mechanisms of YTHDC2 in Non-Tumor Respiratory Diseases

Beyond its established oncogenic roles, the m^6^A reader YTHDC2 orchestrates immune microenvironment remodeling in non-tumor respiratory diseases, primarily through the regulation of macrophage polarization and senescence [31]. Specifically, cigarette smoke extract significantly suppresses YTHDC2 expression in macrophages. This downregulation drives macrophage senescence and M2-like polarization by regulating ribosomal protein S8 (RPS8), leading to increased secretion of pro-inflammatory cytokines and the formation of a chronic inflammatory niche [31]. Concurrently, YTHDC2 modulates macrophage polarization by antagonizing CDCA4. In contrast to the METTL3/ALKBH5-mediated m^6^A modification that drives CDCA4 overexpression and M2 polarization, YTHDC2 specifically recognizes the modified transcript to inhibit CDCA4 expression. This mechanism shifts the balance towards an M1 phenotype, effectively alleviating pathological progression [30]. Beyond these immune mechanisms, accumulating evidence implicates YTHDC2 in broader inflammatory and toxic lung injuries. In acute respiratory distress syndrome (ARDS), a heterogeneous form of lung injury, YTHDC2 mRNA expression is significantly downregulated in a lipopolysaccharide (LPS)-induced murine model. This correlation suggests that YTHDC2 may modulate the inflammatory and pathological processes of ARDS, although direct mechanistic evidence remains lacking [32]. Similarly, while YTHDC2 single-nucleotide polymorphisms (SNPs) do not confer susceptibility to pulmonary tuberculosis (PTB), YTHDC2 mRNA levels are significantly reduced in peripheral blood mononuclear cells (PBMCs) from PTB patients. This reduction highlights its potential as an auxiliary biomarker pending further mechanistic validation [33]. Additionally, environmental toxicants such as cadmium progressively downregulate YTHDC2 expression across successive passages of bronchial epithelial cells exposed to cadmium, implicating YTHDC2 in cadmium-triggered cellular injury pathways [34]. Collectively, these findings underscore YTHDC2 as a critical regulator and potential therapeutic target for modulating immune dysregulation and cellular injury in non-tumor respiratory diseases.

## 4. Multifaceted Roles of YTHDC2 in Digestive System Diseases

Current evidence indicates that YTHDC2 contributes to the pathogenesis and progression of multiple digestive system diseases, highlighting it as a potential therapeutic target.

### 4.1. Mechanisms of YTHDC2 in Digestive System Tumors

Aberrant YTHDC2 expression is closely implicated in esophageal cancer progression. Notably, YTHDC2 is significantly downregulated in esophageal cancer tissues relative to adjacent normal tissues [35]. Consistent with this, genetic knockdown of YTHDC2 in esophageal squamous cell carcinoma (ESCC) cells promotes proliferation and accelerates disease progression [36]. Conversely, Zhao et al. [37] reported contradictory findings, demonstrating that YTHDC2 is upregulated in esophageal cancer, where its elevated expression correlates with advanced tumor stage and poor prognosis. These discrepancies highlight the context-dependent complexity of YTHDC2 in esophageal cancer, necessitating further systematic investigations to establish its potential as a reliable therapeutic target.

Beyond esophageal malignancies, YTHDC2 functions as an oncogene in gastric cancer. It is overexpressed, driving tumor cell proliferation, migration, and invasion, and correlates with poor prognosis [38,39]. Mechanistically, YTHDC2 recognizes the m^6^A modification within the 5ʹ untranslated region of Yes-associated protein (YAP) mRNA, thereby enhancing YAP translation efficiency without altering mRNA abundance. Reciprocally, the YAP/TEA domain transcription factor (YAP/TEAD) complex directly binds the YTHDC2 promoter at positions −843 to −831 to activate its transcription [39]. Together, these reciprocal interactions establish a YTHDC2/YAP positive feedback loop that accelerates gastric cancer initiation and progression. Furthermore, genetic variations in YTHDC2 contribute to targeted therapy resistance. In gastrointestinal stromal tumors (GIST), the specific YTHDC2 rs1833678 T > C single-nucleotide polymorphism is significantly associated with shorter progression-free survival, exhibiting a hazard ratio of 2.87, suggesting its involvement in mediating secondary imatinib resistance. Moreover, a cumulative genetic risk score based on m^6^A pathway variants, including YTHDC2, strongly correlates with disease progression. Collectively, these findings indicate that YTHDC2 polymorphisms may underlie inter-patient prognostic heterogeneity in imatinib-treated GIST and serve as potential biomarkers for predicting secondary resistance [40].

YTHDC2 plays a complex and context-dependent role in hepatocellular carcinoma (HCC) [41]. In a tumor-promoting context, YTHDC2 counteracts the tumor-suppressive function of the long noncoding RNA SPATA41–202. Although SPATA41–202 inhibits HCC tumorigenicity and metastasis in vivo and in vitro, YTHDC2 binds and destabilizes this transcript in an m^6^A-dependent manner, thereby reducing its expression and accelerating tumor progression [42]. Conversely, YTHDC2 also exhibits tumor-suppressive properties under specific conditions. Indetail, YTHDC2 synergizes with methyltransferase-like 14 (METTL14) to recognize METTL14-mediated m^6^A modifications on RPLP2 transcripts. This interaction decreases RPLP2 mRNA stability, ultimately inhibiting the proliferation, migration, and invasion of HCC cells [43]. Furthermore, YTHDC2 facilitates autophagy protein 5 (ATG5) mRNA translation in an m^6^A-dependent manner, thereby inducing autophagic ferroptosis and further restricting HCC progression [9].

Colorectal cancer (CRC) ranks as the third most common malignancy globally, characterized by its prolonged asymptomatic phase and unfavorable prognosis [44,45]. In vitro experiments utilizing colorectal cancer cell lines (HCT116, HCT15) demonstrated that knockdown of YTHDC2 promotes cellular proliferation and migration. Subsequent single-cell analysis revealed an epithelial subpopulation with high YTHDC2 expression exhibited elevated cuproptosis scores [46], indicating YTHDC2 involvement in copper-induced cell death pathways in CRC. Furthermore, YTHDC2 induces 5-fluorouracil (5-FU) chemoresistance in colorectal cancer through endoplasmic reticulum (ER) stress and stress granule assembly [47]. The further mechanistic investigation reveals that knockdown of YTHDC2 in human colorectal cancer cells impairs the recognition and binding to m^6^A modifications on LIM domain kinase 1 (LIMK1) mRNA, thereby increasing LIMK1 transcript stability and subsequently increasing its protein expression. This elevated LIMK1 expression further promotes the phosphorylation of Eukaryotic translation initiation factor 2 subunit alpha (eIF2α), which modulates ER stress and facilitates stress granule formation, ultimately leading to 5-FU chemoresistance [47]. Conversely, a recent study [48] reported that elevated YTHDC2 expression is associated with poor prognosis in CRC. Evaluation of immune infiltration indicated that YTHDC2 positively correlates with both immunosuppressive and immune agonistic signals, implying its role in CRC pathogenesis via immunomodulatory mechanisms. Thus, the regulatory impact of YTHDC2 in CRC appears to be heterogeneous, likely stemming from interpatient variability and differences in clinical stages. These observations identify YTHDC2 as a potential therapeutic target, providing a strong rationale for the advancement of combined immunotherapeutic and targeted interventions for CRC.

Altogether, YTHDC2 exhibits highly heterogeneous functions in digestive system tumors. While it acts as an oncogene in gastric and hepatocellular carcinomas, evidence in colorectal cancer points to a tumor-suppressive role linked to chemoresistance. Conversely, reports in esophageal cancer remain contradictory. Consequently, the net effect of YTHDC2 in these malignancies is difficult to generalize, appearing strictly dependent on tumor type and specific context.

### 4.2. Mechanisms of YTHDC2 in Non-Tumor Diseases of the Digestive System

**Figure 2:**
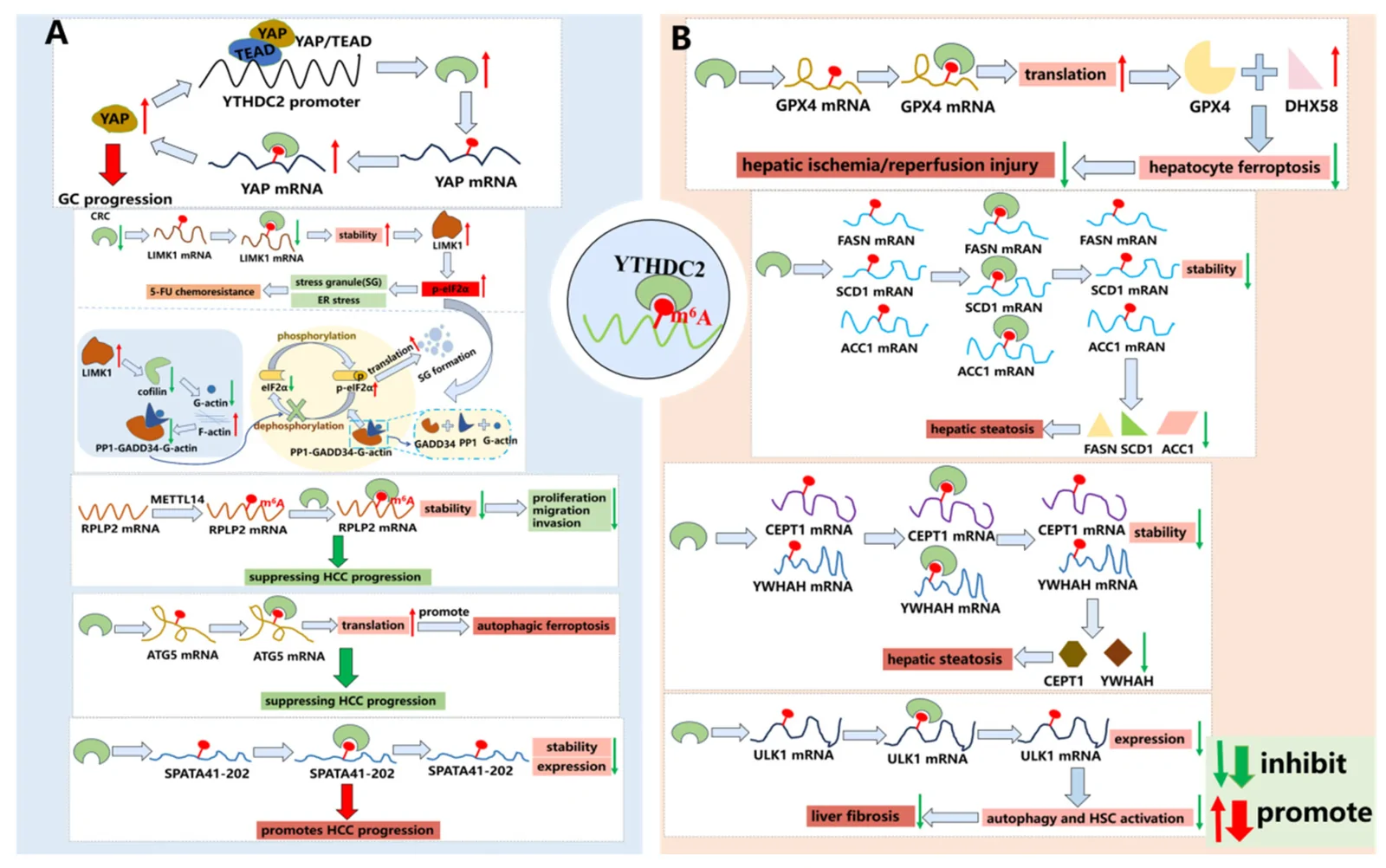
m^6^A-Dependent Mechanisms of YTHDC2 in Digestive System Diseases. (**A**) Molecular mechanism diagram illustrating how YTHDC2 exerts pro-or anti-cancer effects in digestive system tumors (e.g., colorectal cancer, gastric cancer, and hepatocellular carcinoma) by recognizing m^6^A modifications on specific target gene mRNAs. (**B**) Detailed mechanism diagram showing how YTHDC2 inhibits disease progression in non-neoplastic digestive system disorders (e.g., hepatic ischemia/reperfusion injury, liver fibrosis, etc.) by recognizing m^6^A modifications on specific target mRNAs.

YTHDC2 orchestrates a complex regulatory network within the liver, influencing the progression of metabolic disorders, ischemic injury, and fibrosis through distinct m^6^A-dependent mechanisms. In detail, in non-alcoholic fatty liver disease (NAFLD), YTHDC2 expression is significantly downregulated and negatively correlates with steatosis percentage [49]. YTHDC2 suppresses steatosis by enhancing lipid-metabolism-related genes, including choline/ethanolamine phosphotransferase 1 (CEPT1) and tyrosine 3-monooxygenase/tryptophan 5-monooxygenase activation protein, eta polypeptide (YWHAH). Notably, PM_2.5_ exposure downregulates YTHDC2, impairing its recognition of m^6^A sites on these target mRNAs. This reduces transcript stability and suppresses protein expression, thereby promoting steatosis [50]. Consistent with a protective role, YTHDC2 is downregulated in obese livers, and its hepatic overexpression ameliorates steatosis and insulin resistance. Mechanistically, YTHDC2 binds m^6^A-modified mRNAs encoding lipogenic enzymes such as fatty acid synthase (FASN), stearoyl-CoA desaturase 1 (SCD1), and acetyl-CoA carboxylase 1 (ACC1), reducing their stability and expression to attenuate steatosis [5]. Conversely, a study on type 2 diabetes reported elevated YTHDC2 levels, suggesting a distinct involvement in hepatic injury [51]. Beyond metabolic disease, YTHDC2 ameliorates hepatic ischemia/reperfusion (I/R) injury by inhibiting ferroptosis. It promotes the m^6^A-dependent translation of GPX4 mRNA, enhancing the interaction between GPX4 and DHX58 to prevent hepatocyte death [52]. In liver fibrosis, YTHDC2 inhibits hepatic stellate cell (HSC) activation by recognizing m^6^A sites on Unc-51 like autophagy activating kinase 1 (ULK1) mRNA. This interaction reduces ULK1 levels, suppresses autophagy, and mitigates fibrogenesis [53]. Thus, YTHDC2 represents a promising therapeutic target for hepatic steatosis, I/R injury, and fibrosis. The multifaceted roles of YTHDC2 in Digestive System Diseases are summarized in Table 2. And m^6^A-dependent mechanisms of the reader YTHDC2 in Digestive System Diseases were represented in Figure 2. However, current studies are largely confined to preclinical models with limited insight into upstream regulatory networks. Future studies must elucidate the precise mechanisms governing YTHDC2 expression and validate its therapeutic potential clinically to bridge the gap between bench and bedside.

## 5. Multifaceted Roles of YTHDC2 in Renal Diseases

Diabetic nephropathy (DN) stands as one of the most devastating chronic microvascular complications of diabetes mellitus, characterized by a high prevalence and substantial morbidity and mortality. Studies have revealed significantly elevated levels of YTHDC2 expression in patients with DN compared with normal controls, suggesting a potential critical role in disease pathogenesis [54]. Furthermore, YTHDC2 interacts with the long non-coding RNA HOXB3AS to mitigate high-glucose-induced podocyte injury and delay the progression of diabetic nephropathy. Mechanistically, HOXB cluster antisense RNA 3 (HOXB-AS3) functions as a molecular decoy, competitively binding to YTHDC2 and preventing its interaction with Sirtuin 1 (SIRT1) mRNA. This sequestration inhibits the YTHDC2-mediated degradation of SIRT1 transcripts, resulting in elevated SIRT1 expression and subsequent protection against podocyte damage [55]. Mechanistically, YTHDC2 destabilizes SIRT1 mRNA under high-glucose conditions, thereby exacerbating podocyte injury. In contrast, HOXB-AS3 competitively binds to YTHDC2 to block this degradation, exerting a protective effect. In the context of obstructive megaureter, YTHDC2 mRNA levels are significantly elevated compared to primary vesicoureteral reflux tissues [56], suggesting a correlative association that requires further mechanistic validation. In membranous nephropathy, high YTHDC2 expression is predominantly associated with metabolic pathways, whereas low expression is primarily linked to chemokine signaling. Furthermore, YTHDC2 levels exhibit a negative correlation with the abundance of most immune cell populations, implying a potential regulatory role for YTHDC2 in modulating immune cell infiltration [57]. However, this evidence is currently correlative, and YTHDC2 is identified as one of several potential diagnostic biomarkers rather than a proven pathogenic driver.

Altogether, YTHDC2 exhibits highly heterogeneous functions in renal diseases. While it acts as a critical pathogenic driver in diabetic nephropathy by promoting SIRT1 mRNA degradation, evidence in obstructive megaureter and membranous nephropathy points to a role primarily as a diagnostic biomarker. In these non-diabetic conditions, YTHDC2 expression correlates with metabolic states or immune cell depletion, yet causality remains unproven. Consequently, the role of YTHDC2 is difficult to generalize, appearing strictly dependent on the specific disease context.

## 6. Multifaceted Roles of YTHDC2 in Reproductive System Diseases

YTHDC2 serves as a pivotal regulatory factor with significant biological functions in reproductive system diseases such as ovarian cancer and prostate cancer, while also playing an essential role in meiotic progression and germ cell development [16,58,59,60,61,62,63,64,65,66].

### 6.1. The Role and Mechanisms of YTHDC2 in Reproductive System Tumors

Ovarian cancer remains a highly lethal gynecological malignancy. Research indicates that YTHDC2 expression is downregulated in ovarian cancer and inversely correlates with unfavorable prognosis. Bioinformatic analyses identified filamin A (FLNA) as a potential downstream target containing m^6^A modification sites, with immunohistochemical analysis corroborating a positive correlation between YTHDC2 and FLNA expression in epithelial ovarian cancer (EOC) tissues. However, direct binding between YTHDC2 and FLNA mRNA requires experimental validation [58]. Conversely, Zhu et al. [59] reported that elevated YTHDC2 levels positively correlated with poor prognosis. These discrepancies may be attributable to interpatient heterogeneity and variations in disease stage. Regarding prostate cancer, a major global health concern, YTHDC2 is significantly upregulated. Functionally, YTHDC2 overexpression enhances the proliferative, migratory, and invasive capacities of prostate cancer cells [60]. Furthermore, YTHDC2 counteracts the tumor-suppressive effects of Circular RNA midline-1 (circMID1) silencing. Mechanistically, elevated YTHDC2 promotes IGF-1R protein synthesis, subsequently activating the AKT signaling pathway. This cascade potentiates proliferative, migratory, invasive, and glycolytic phenotypes in prostate carcinoma cells [61].

In summary, while YTHDC2 functions as a robust oncogenic driver in prostate cancer through the IGF-1R/AKT axis, its role in ovarian cancer remains ambiguous due to discrepant clinical data and a lack of direct mechanistic validation, highlighting the need for further stratified studies in reproductive malignancies.

### 6.2. Mechanisms of YTHDC2 in Non-Tumor Diseases of the Reproductive System

Research has identified that YTHDC2 interacts with multiple proteins present in RNA granules to promote the timely progression of the meiotic program [16]. The underlying mechanism likely involves YTHDC2 collaborating with RNA granule components to recruit target RNAs into these granules. Subsequently, depending on the specific granule composition, the recruited RNAs are selectively degraded, translationally silenced, or stored for future use. This process facilitates the precise regulation of RNA expression during transitions between different cellular states in germ cells [16]. It has been reported that the RNA-binding complex (meiosis-specific coiled-coil domain-containing protein (MEIOC)-YTHDC2-RNA-binding motif protein 46 (RBM46))-mediated mRNA decay enhances the ability of spermatogenic cells to initiate meiosis. The mechanism involves the MEIOC collaborating with YTHDC2 and RBM46 to degrade mitotic target mRNAs (including the transcriptional repressors E2F transcription factor 6 (E2f6) and MAX gene associated (Mga)) in spermatogonia. This process confers upon spermatogenic cells the competence to respond to retinoic acid, thereby activating the meiotic transcriptional regulators stimulated by retinoic acid 8 (STRA8)-MEIOSIN, upregulating the expression of meiosis-and cell cycle-related genes, and driving the meiotic G1/S phase transition [62].

Adenosine deaminase domain-containing protein 2 (ADAD2) is a testis-specific protein. Studies have demonstrated that YTHDC2 interacts with ADAD2 and participates in murine spermatogenesis [63]. Research has identified the involvement of the YTHDC2/cyclin B2 (CCNB2) signaling pathway in manganese-induced reproductive toxicity. Notably, overexpression of YTHDC2 was found to alleviate the deleterious effects of Mn exposure. The underlying mechanism involves YTHDC2 upregulation leading to increased levels of CCNB2, which in turn reduces cell cycle arrest and ameliorates the reproductive toxicity observed following manganese exposure [64]. Studies indicate that in male mouse models of malformations induced by cadmium and high-fat diet, YTHDC2 expression is significantly downregulated in testicular tissues compared with normal controls, suggesting its potential involvement in the pathogenesis of reproductive organ abnormalities under these conditions [65].

Studies have identified pathogenic variants in YTHDC2 among women with Premature Ovarian Insufficiency (POI), presenting with absent puberty and primary amenorrhea. However, these YTHDC2 mutants did not alter the stability or splicing of YTHDC2 transcripts in peripheral leukocytes. Further investigations demonstrate that YTHDC2 is expressed in developing human fetal ovaries and is upregulated in meiotic germ cells, supporting its role as a critical regulator of human meiosis [66].

**Figure 3:**
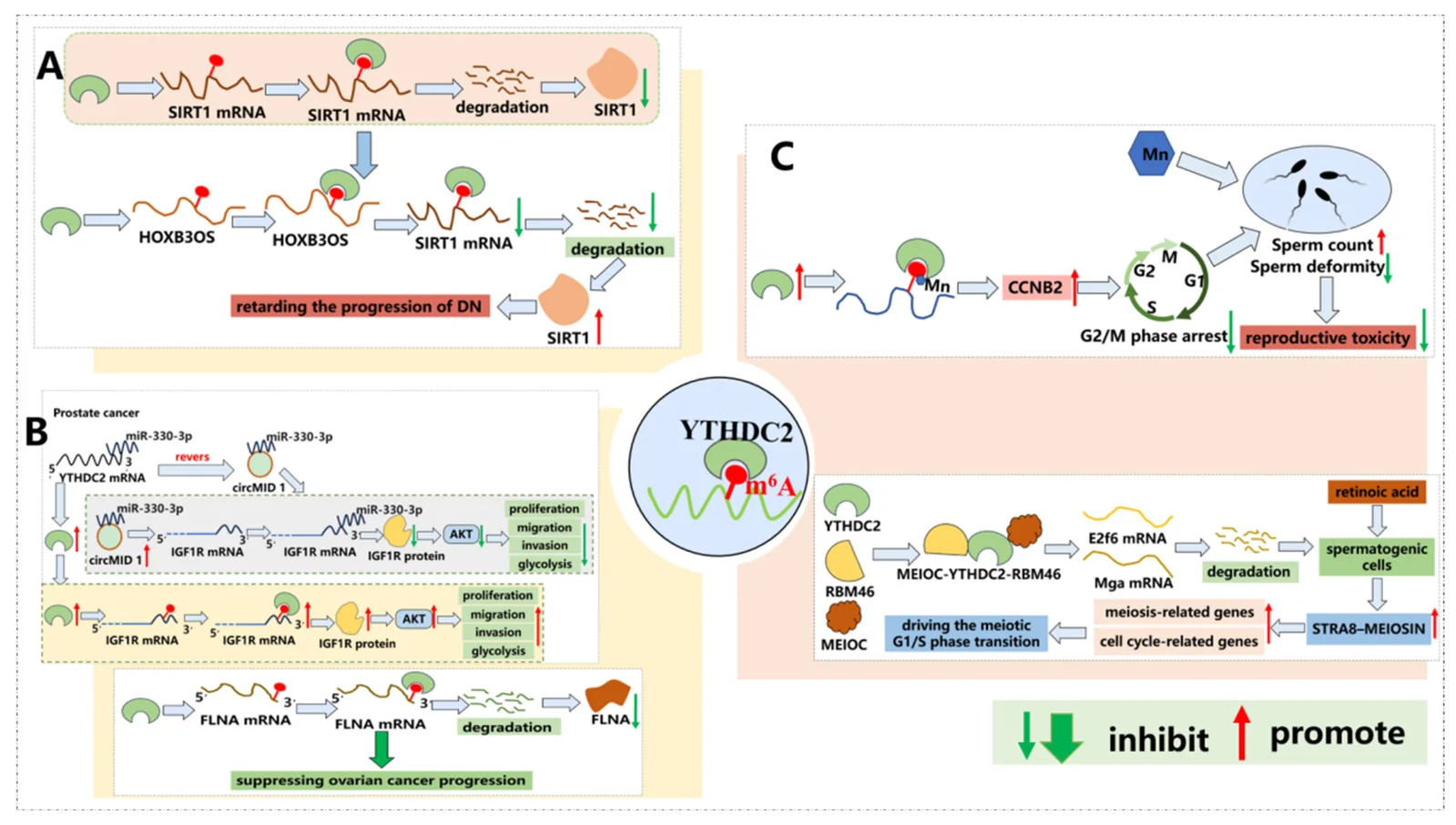
m^6^A-dependent mechanisms of the reader YTHDC2 in Renal Diseases and Reproductive System Diseases. (**A**) YTHDC2 binds lncRNA HOXB3OS to upregulate SIRT1 and delay diabetic nephropathy. (**B**) YTHDC2 exerts oncogenic effects in prostate cancer and tumor-suppressive functions in ovarian cancer by targeting distinct mRNAs. (**C**) YTHDC2 facilitates germ cell meiosis via the MEIOC-YTHDC2-RBM46 complex and mitigates manganese-induced reproductive toxicity through CCNB2 upregulation.

Collectively, YTHDC2 emerges as a pivotal rheostat for meiotic fidelity, bridging genetic stability and environmental responsiveness. Pathogenic variants directly precipitate ovarian insufficiency, whereas external stressors like heavy metals and diet compromise its function in males. This distinct vulnerability highlights YTHDC2 as a critical node in reproductive homeostasis. Future investigations must elucidate how environmental signals modulate this regulatory axis, a mechanism currently lacking detailed characterization. The multifaceted roles of YTHDC2 in Renal Diseases and Reproductive System Diseases are summarized in Table 3. And m^6^A-dependent mechanisms of the reader YTHDC2 in Renal Diseases and Reproductive System Diseases are represented in Figure 3.

## 7. Multifaceted Roles of YTHDC2 in Endocrine System Diseases

YTHDC2 has emerged as a candidate molecular target in thyroid cancer, type 1 diabetes (T1D), type 2 diabetes (T2D), and their associated complications, making elucidation of its underlying mechanisms particularly important [67,68,69,70,71,72,73].

### 7.1. Mechanisms of YTHDC2 in Endocrine System Tumors

Papillary thyroid carcinoma (PTC) represents the most prevalent endocrine malignancy, characterized by a steadily rising global incidence. Notably, YTHDC2 is downregulated in PTC tissues, whereas its overexpression suppresses proliferation and promotes apoptosis. Mechanistically, YTHDC2 orchestrates anti-tumor effects through two complementary pathways: modulating apoptotic regulators by upregulating pro-apoptotic factors (cleaved caspase-3 and Bax) while downregulating Bcl-2, and attenuating AKT signaling via enhanced K63-linked deubiquitination [67]. Furthermore, YTHDC2 synergizes with methyltransferase-like 16 (METTL16) to curb PTC progression by modulating lipid metabolism through the lipogenic gene SCD1. Mechanistically, YTHDC2 triggers the m^6^A-dependent decay of SCD1 mRNA, effectively restraining tumor advancement [68].

### 7.2. Mechanisms of YTHDC2 in Non-Tumor Endocrine Diseases

T1D is a severe disorder that typically necessitates intensive insulin therapy. A genetic study in a Chinese Han population identified intronic variants in Proline Rich Coiled-Coil 2A (PRRC2A) (rs2260051, rs3130623) and YTHDC2 (rs1862315) that confer susceptibility to T1D. Notably, single-nucleotide polymorphisms (SNPs) in the non-coding regions of both genes correlate with disease risk. Mechanistically, the PRRC2A-YTHDC2[T] haplotype appears to modulate PRRC2A mRNA expression, thereby disrupting immune homeostasis. These findings elucidate the genetic basis of T1D susceptibility and offer insights into its pathogenesis [69]. T2D and its complications continues to rise globally. Employing linkage disequilibrium score regression and high-definition likelihood methods, genetic correlation analysis demonstrated that elevated YTHDC2 expression is positively associated with susceptibility to T2D [70]. Furthermore, circRNA microarray profiling identified a novel circular RNA, circYTHDC2, whose stability is modulated by YTHDC2 through m^6^A modification. Knockdown of circYTHDC2 significantly attenuates vascular smooth muscle cell (VSMC) proliferation and invasion. Mechanistically, circYTHDC2 negatively regulates ten-eleven translocation 2 (TET2), thereby driving the VSMC phenotypic transition to a synthetic state under T2D-associated high-glucose conditions [71]. Beyond its role in VSMC dysfunction, circYTHDC2 exerts broader regulatory functions. Biogenetically, it originates from exons 13–18 of the YTHDC2 gene via backsplicing and is highly conserved across species [72]. Notably, circYTHDC2 directly interacts with YTHDC2 and undergoes m^6^A-dependent positive regulation by the protein, implying a potential self-regulatory feedback loop [73]. Beyond TET2, circYTHDC2 targets Stimulator of Interferon Genes (STING) for degradation via ubiquitination in fish models, thereby dampening antiviral innate immunity [72]. Moreover, circYTHDC2 is significantly upregulated in HCC tissues, serum, and cell lines, and its elevated levels correlate with adverse clinicopathological features, including advanced Barcelona Clinic Liver Cancer staging, larger tumor volume, and vascular invasion. Notably, circYTHDC2 demonstrates high diagnostic accuracy in both tissue and serum samples, positioning it as a stable and clinically viable biomarker for HCC. Collectively, these findings reveal the multifaceted regulatory roles of circYTHDC2 beyond the cardiovascular system [73].

## 8. Multifaceted Roles of YTHDC2 in Immune System Diseases

YTHDC2 attenuates the innate antiviral response in virus-infected macrophages by recruiting the exonuclease interferon-stimulated exonuclease gene 20 (ISG20) to promote interferon-β (IFN-β) mRNA degradation, thereby ensuring proper termination of the immune response at the late stage of infection [10]. While the viral nuclease SRY-related HMG-box (SOX) induces global mRNA degradation during Kaposi’s sarcoma-associated herpesvirus (KSHV) lytic infection, YTHDC2 selectively safeguards interleukin-6 (IL-6) transcripts from vSOX-mediated decay. This m^6^A-dependent protection mechanism ensures sustained IL-6 expression, subverting the host shutoff response [74]. Concurrently, YTHDC2 promotes immune evasion by *Talaromyces marneffei*. Mechanistically, upregulated YTHDC2 recognizes m^6^A modifications on Toll-like receptor 2 (TLR2) mRNA, leading to TLR2 downregulation and subsequent attenuation of pro-inflammatory cytokine secretion (TNF-α and IL-1β) [75].

Rheumatoid arthritis (RA) is a chronic autoimmune disorder characterized by systemic joint inflammation, leading to irreversible joint destruction and functional disability. Studies indicate that YTHDC2 is downregulated in rheumatoid arthritis fibroblast-like synoviocytes (RA-FLS), and its restoration significantly inhibits cell proliferation and invasion. Mechanistically, YTHDC2 accelerates the degradation of amphoterin-induced gene and open reading frame 2 (AMIGO2) mRNA, thereby attenuating AMIGO2-mediated pathogenic behaviors in RA-FLS [76]. Collectively, these findings indicate that YTHDC2 plays a protective role in RA-FLS activation via the METTL3-m^6^A-YTHDC2-AMIGO2 axis, functioning as a critical negative regulator.

Uveitis is a sight-threatening disorder driven by dysregulated immune responses. Studies indicate that YTHDC2 significantly attenuates the severity of experimental autoimmune uveitis (EAU). Mechanistically, YTHDC2 enhances the stability of absent, small, or homeotic discs 1-like (ASH1L) mRNA in an m^6^A-dependent manner, thereby promoting ASH1L expression. This subsequently downregulates the expression of interleukin-17 (IL-17) and the IL-23 receptor, ultimately dampening the pathogenic response of T helper 17 (Th17) cells [77]. Consistently, Systemic sclerosis (SSc) is a chronic autoimmune fibrotic disorder characterized by fibrosis of the skin and vascular system. A comparative study of mRNA expression levels in 61 SSc patients and 41 healthy controls revealed significantly downregulated YTHDC2 expression in the patient cohort, implicating its potential role in the pathogenesis of SSc [78].

In summary, YTHDC2 exhibits bidirectional regulatory roles across distinct immune contexts. In infectious settings, it modulates immune responses, while in autoimmune disorders like rheumatoid arthritis, uveitis, and systemic sclerosis, it functions as a protective suppressor, with its downregulation exacerbating pathological progression. Despite these insights, the molecular switches dictating its substrate selectivity in different microenvironments remain largely undefined. Future research should focus on elucidating these tissue-specific regulatory networks to provide a theoretical basis for precision therapies targeting YTHDC2.

## 9. Multifaceted Roles of YTHDC2 in Other Systemic Diseases

In smokers with coronary artery disease (CAD), tobacco exposure downregulates YTHDC2 under hypoxic conditions, as evidenced by analyses of peripheral blood samples from CAD patients and validated in oxygen-glucose deprivation (OGD)-treated Human Umbilical Vein Endothelial Cells (HUVECs) and FMC84 mouse cardiomyocytes, thereby accelerating disease progression. Notably, elevated YTHDC2 levels correlate with increased infiltration of CD4^+^ and activated CD8^+^ T cells, whereas reduced expression associates with enhanced macrophage accumulation. Collectively, these findings suggest that dysregulated YTHDC2 may serve as a critical nexus linking epitranscriptomic modifications to immune infiltration in smoking-related CAD [79]. Similarly, in septic cardiomyopathy, YTHDC2 is differentially expressed in LPS-induced injury models and promotes cardiomyocyte apoptosis by directly binding to target mRNAs, thereby activating the NF-κB pathway [80]. These findings establish a key regulatory role for YTHDC2 in the pathogenesis of both smoking-related CAD and septic cardiomyopathy.

Beyond the cardiovascular system, YTHDC2 exerts protective functions in tissue regeneration and retinal homeostasis. In human adipose-derived stem cells (hASCs), YTHDC2 governs osteogenesis through the AK311120-DHX9-YTHDC2 axis: it facilitates the translation of mitogen-activated protein kinase kinase 7 (MAP2K7) and activates the downstream JNK signaling cascade to drive osteogenic differentiation [81]. In the retina, YTHDC2 deficiency exacerbates hereditary retinal dystrophy by diminishing the translational efficiency of Protein phosphatase with EF-hand domain 2 (PPEF2) and Phosphodiesterase 6B (PDE6B), culminating in visual dysfunction and photoreceptor degeneration [82]. Additionally, YTHDC2 is significantly upregulated in the aqueous humor of pseudoexfoliation glaucoma patients, implicating its potential involvement in the pathogenesis of this condition [83].

Furthermore, in preeclampsia, YTHDC2 interacts with the lncRNA AC092100.1 to enhance VEGFA expression in human umbilical vein endothelial cells (HUVECs), thereby promoting angiogenesis and alleviating disease progression. This suggests a protective role through the AC092100.1/YTHDC2/VEGFA axis [84].

In summary, YTHDC2 exhibits bidirectional regulatory roles across distinct physiological contexts. In cardiovascular pathologies, it promotes disease progression by modulating immunity and apoptosis, whereas in tissue regeneration and retinal homeostasis, it acts as a critical protector. However, the molecular switches governing its substrate selectivity across different microenvironments remain largely undefined. Future investigations should elucidate these tissue-specific regulatory networks to establish a theoretical basis for precision therapies targeting YTHDC2.

## 10. Conclusions and Perspectives

As a pivotal member of the YTH family, YTHDC2 has emerged as a critical player in both neoplastic and non-neoplastic diseases. Current evidence underscores its essential role in orchestrating diverse pathological processes via RNA metabolism regulation. In neoplastic diseases, YTHDC2 exhibits context-dependent functions in malignancies such as lung cancer and hepatocellular carcinoma, suggesting tissue- and stage-specific regulatory modes that warrant further dissection of the underlying m^6^A-dependent molecular pathways. Similarly, in non-neoplastic conditions, YTHDC2 demonstrates heterogeneous roles: it exerts protective effects in hepatic steatosis, ischemia/reperfusion injury, and preeclampsia, whereas it mediates pathogenic progression in diabetic nephropathy. However, for conditions like membranous nephropathy and myocardial injury, current evidence remains largely correlative and requires rigorous mechanistic validation.

It is noteworthy that the studies cited herein vary in the strength of evidence. As summarized in Supplementary Table S1, we have categorized the evidence into three levels based on the availability of animal models, functional assays, m^6^A binding/modification validation, and mechanistic interrogation (e.g., MeRIP/RIP, site-directed mutagenesis, and rescue experiments). Studies with complete mechanistic validation are designated as Grade I, those with partial functional characterization but lacking complete mechanistic dissection as Grade II, and those relying primarily on expression correlations or clinical associations as Grade III. Consequently, YTHDC2 should currently be regarded as an emerging candidate regulator whose clinical relevance as a diagnostic marker or therapeutic target varies substantially across disease contexts.

Despite its therapeutic promise, clinical translation faces significant obstacles. First, the infertility observed in *Ythdc2*-knockout mice indicates an indispensable role in meiosis, raising concerns regarding potential reproductive toxicity upon systemic inhibition. Second, the context-dependent, often opposing effects of YTHDC2 across different tumor types complicate the prediction of therapeutic outcomes. Third, the absence of clinical-grade specific inhibitors limits immediate intervention; targeting upstream m^6^A writers or erasers may offer alternatives but lacks specificity. Future research should prioritize tissue-specific delivery systems, conditional targeting strategies, and long-term safety monitoring. Integrating spatial transcriptomics and artificial intelligence could further enhance the spatiotemporal understanding of YTHDC2 within tissue microenvironments. Collectively, elucidating the precise mechanistic landscape of YTHDC2 will not only advance our comprehension of m^6^A-dependent pathogenesis but also pave the way for novel precision therapies.

## Figures and Tables

**Table 1:** Multifaceted Roles of YTHDC2 in Respiratory Tumors.

Diseases	Subjects	Target RNAs	Functional Roles	Refs
Nasopharyngeal carcinoma	Patient, cell (CNE2, CNE2-IRR, HK1, HK1-IRR), nude mouse	IGF-1R mRNA	YTHDC2 upregulation enhances IGF-1R mRNA translation (m^6^A-dependent), activating IGF-1R/AKT/S6 axis to confer radioresistance in nasopharyngeal carcinoma.	[24]
Non-small cell lung cancer (NSCLC)	Patient, cell (H1299, BEAS-2B, HUVECs), BALB/c nude mouse	ZNRD1-AS1 lncRNA	YTHDC2 recognizes m^6^A-modified ZNRD1-AS1, enhancing its stability and activating the miR-942/TNS1 axis to promote malignant proliferation and angiogenesis in NSCLC.	[25]
	Patient, cell (BEAS-2B, NCI-H1299, HCC-827, NCI-H358, A549), BALB/c nude mouse	CALML3-AS1 lncRNA	YTHDC2 cooperates with ALKBH5 to regulate m^6^A-modified lncRNA CALML3-AS1, which recruits EZH2 to epigenetically silence BTNL9, driving NSCLC progression.	[26]
Lung cancer (LC)	Cell (A549, H1299, HepG2, HeLa, HUVECs), mouse (C57BL/6)	VEGFA mRNA	YTHDC2 recognizes m^6^A at VEGFA 5′UTR IRES-A, recruiting the eIF4GI complex to enhance cap-independent translation, stimulating angiogenesis and lung tumor growth.	[27]
Lung adenocarcinoma (LUAD)	Patient, cell (A549, H1573, HBE), BALB/c nude mouse	MRPL12 mRNA	YTHDC2 destabilizes MRPL12 mRNA in an m^6^A-dependent manner, thereby repressing LUAD cell growth and mobility in vitro and suppressing tumor growth in vivo.	[28]
	Cell (A549, H1299, H1650, H1975), nude mouse	IFIT/OAS family mRNAs	YTHDC2 downregulation decreases IFIT/OAS expression, enhancing oncogenic exosome content and facilitating intercellular communication in lung adenocarcinoma.	[29]
	Patient, cell (NCI-H157, NCI-H1299, NCI-H1975, BEAS-2B), mouse (LLC)	CDCA4 mRNA	YTHDC2 overexpression destabilizes m^6^A-modified CDCA4 mRNA, increasing the M1/M2 ratio and suppressing tumor growth by fostering an anti-tumor immune microenvironment.	[30]

**Table 2:** Multifaceted Roles of YTHDC2 in Digestive System Diseases.

Diseases	Subjects	Target RNAs	Functional Roles	Refs
Gastric cancer	Patient, cell (AGS and HGC-27), nude mouse	YAP mRNA	YTHDC2 overexpression recognizes m^6^A in YAP 5′UTR, enhancing YAP translation. The YAP/TEAD complex activates YTHDC2 transcription, creating a positive feedback loop driving progression.	[39]
Hepatocellular carcinoma	Patient, cell (LO2, HCCLM3, MHCC97H, 97L, HepG2, Huh7, SMMC-7721,7402 and Hep3B), BALB/c nude mouse	SPATA41–202 lncRNA	YTHDC2 binds and destabilizes m^6^A-modified SPATA41–202 lncRNA, counteracting its tumor-suppressive function and thereby accelerating hepatocellular carcinoma progression.	[42]
	Patient, cell (L02, MHCC97H, SNU182, Huh7, HCCLM3)	RPLP2 mRNA	YTHDC2 synergizes with METTL14 to recognize m^6^A on RPLP2 transcripts, decreasing RPLP2 mRNA stability and inhibiting HCC cell proliferation and invasion.	[43]
	Patient, cell (Huh-7, HepG2), BALB/c nude mouse	ATG5 mRNA	YTHDC2 facilitates ATG5 mRNA translation in an m^6^A-dependent manner, thereby inducing autophagic ferroptosis and restricting hepatocellular carcinoma progression.	[9]
Colorectal cancer	Patient, cell (LoVo, HCT116, HT29, Caco2, RKO, SW480, NCM460, FHC)	LIMK1 mRNA	YTHDC2 knockdown stabilizes m^6^A-modified LIMK1 mRNA, increasing LIMK1 expression, promoting ER stress, stress granule formation, and 5-FU chemoresistance in colorectal cancer.	[47]
Non-alcoholic fatty liver disease (NAFLD)	Cell (HepG2) Mouse (C57BL/6J)	CEPT1, YWHAH mRNAs	PM_2.5_ downregulates YTHDC2, impairing recognition of m^6^A sites on CEPT1/YWHAH mRNAs. This reduces transcript stability and suppresses protein expression, thereby promoting steatosis.	[50]
	Human, HFD, Mouse (db/db and ob/ob)	FASN, SCD1, ACC1 mRNAs	YTHDC2 downregulation impairs recognition of m^6^A sites on lipogenic genes, increasing their stability and expression, thereby promoting hepatic steatosis.	[5]
Hepatic ischemia/reperfusion (I/R) injury	Patient, mouse (C57BL/6)	GPX4 mRNA	YTHDC2 promotes GPX4 mRNA translation (m^6^A-dependent), enhancing the GPX4-DHX58 interaction to prevent hepatocyte ferroptosis and ameliorate hepatic ischemia/reperfusion injury.	[52]
Liver fibrosis	Cell (HSC JS-1), mouse (C57BL/6)	ULK1 mRNA	YTHDC2 inhibits hepatic stellate cell activation by recognizing m^6^A sites on ULK1 mRNA, reducing ULK1 levels, suppressing autophagy, and mitigating fibrogenesis.	[53]

**Table 3:** Multifaceted Roles of YTHDC2 in Renal Diseases and Reproductive System Diseases.

Diseases	Subjects	Target RNAs	Functional Roles	Refs
Diabetic nephropathy (DN)	Cell (MPC5) mouse (C57BL/KsJ db/db, C57BL/KsJ db/m)	SIRT1 mRNA	YTHDC2 upregulation mediates SIRT1 mRNA degradation (m^6^A-dependent) under high glucose, causing podocyte injury; LncRNA HOXB3AS competitively binds YTHDC2 to protect SIRT1.	[55]
Ovarian cancer	Epithelial ovarian cancer (EOC) tissues	FLNA mRNA	YTHDC2 is downregulated in ovarian cancer tissues and inversely correlates with prognosis, though contradictory evidence exists; FLNA is a potential target.	[58]
Prostate cancer	Patient, cell (PC-3, DU145, RWPE-1), BALB/c nude mouse	IGF-1R mRNA	YTHDC2 upregulation promotes IGF-1R protein synthesis, activating the AKT pathway to enhance proliferative, migratory, invasive, and glycolytic phenotypes in prostate cancer.	[61]
Meiosis (Mouse)	Mouse (C57BL/6)	E2f6, Mga mRNAs	The MEIOC-YTHDC2-RBM46 complex destabilizes mitotic mRNAs (e.g., E2f6), enabling meiotic G1/S transition via STRA8-MEIOSIN activation in mouse spermatogenic cells.	[62]
Manganese-induced reproductive toxicity	Patient, cell (GC-1), KM mouse	CCNB2 mRNA	YTHDC2 overexpression upregulates CCNB2, reducing cell cycle arrest and thereby alleviating manganese-induced reproductive toxicity in male mouse models.	[64]

## Data Availability

No new data were created or analyzed in this study. Data sharing is not applicable to this article.

## Supplementary Materials

The following supporting information can be downloaded at: https://www.mdpi.com/article/10.3390/cells15161467/s1. Supplementary Table S1. Evidence Level Classification of YTHDC2-Related Studies.
