# Supplementary material for: The m6A Reader YTHDC2: Molecular Mechanisms and Regulatory Networks in Disease Pathogenesis
Source: Cells. 2026 Aug 16;15(16):1467. doi: 10.3390/cells15161467 (PMC13511726; doi:10.3390/cells15161467)
Supplement: Supplementary file 1 [file cells-15-01467-s001.zip › cells-4443472-supplementary.pdf]

Supplementary Table S1. Evidence Level Classification of YTHDC2-Related Studies

Disease/Condition	Ref	Animal Models	Functional Assays	M^6A Binding/ Modification	Mechanistic Validation	Evidence Level
Nasopharyngeal carcinoma	[24]	✓	✓	—	—	II
NSCLC (ZNRD1-AS1)	[25]	✓	✓	✓	—	II
NSCLC (CALML3-AS1)	[26]	✓	✓	✓	✓	I
Lung cancer (VEGFA)	[27]	✓	✓	✓	—	II
LUAD (MRPL12)	[28]	✓	✓	✓	—	II
LUAD (IFIT/OAS)	[29]	—	✓	✓	—	II
LUAD (CDCA4)	[30]	✓	✓	—	—	II
ARDS	[32]	✓	—	—	—	III
Cadmium-induced injury	[34]	✓	✓	✓	—	II
ESCC	[36]	—	✓	—	—	II
Gastric cancer (YAP)	[39]	✓	✓	✓	✓	I
HCC (SPATA41-202)	[42]	✓	✓	✓	—	II
HCC (RPLP2)	[43]	—	✓	✓	—	II
HCC (ATG5)	[9]	✓	✓	✓	✓	I
CRC (LIMK1)	[47]	—	✓	✓	—	II
NAFLD (CEPT1/YWHAH)	[50]	—	✓	✓	—	II
NAFLD	[5]	✓	✓	✓	—	II
Hepatic I/R injury	[52]	✓	—	✓	—	II
Liver fibrosis	[53]	✓	✓	✓	—	II

Diabetic nephropathy	[55]	√	√	—	—	II
Prostate cancer	[61]	√	√	—	—	II
Meiotic regulation	[62]	√	√	√	—	I
Spermatogenesis (ADAD2)	[63]	√	—	√	—	II
Manganese-induced toxicity	[64]	√	√	—	—	II
Cadmium reproductive toxicity	[65]	√	—	—	—	III
Papillary thyroid cancer (CYLD)	[67]	—	√	—	√	II
Papillary thyroid cancer (SCD1)	[68]	—	√	√	—	II
circYTHDC2/VSMC	[71]	—	√	√	—	II
Antiviral immunity	[10]	√	√	√	—	II
KSHV infection	[74]	√	√	√	—	II
Talaromyces marneffei	[75]	√	√	√	—	II
Rheumatoid arthritis	[76]	—	√	—	—	II
Autoimmune uveitis	[77]	√	√	√	—	II
Coronary artery disease	[79]	√	√	—	—	II
Osteogenic differentiation	[81]	—	√	√	—	II
Retinal function	[82]	√	√	√	—	II
Pseudoexfoliation glaucoma	[83]	—	—	√	—	III
Preeclampsia	[84]	√	√	√	—	II
Myocardial injury	[80]	√	√	√	—	II
